# Seasonal variation in the utility of a status signaling system: Plumage ornament predicts foraging success only during periods of high competition

**DOI:** 10.1371/journal.pone.0185584

**Published:** 2017-10-03

**Authors:** Philip S. Queller, Troy G. Murphy

**Affiliations:** Department of Biology, Trinity University, San Antonio, Texas, United States of America; Universite de Lausanne, SWITZERLAND

## Abstract

Status signals allow competitors to assess each other’s resource holding potential and reduce the occurrence of physical fights. Because status signals function to mediate competition over resources, a change in the strength of competition may affect the utility of a status signaling system. Status signals alter competitor behavior during periods of high competition, and thus determine access to resources; however, when competition is reduced, we expect these signals to become disassociated from access to resources. We investigated seasonal changes in status signaling of the male black-crested titmouse (*Baeolophus atricristatus*), a species that experiences substantial changes in population density and competition for food over the annual cycle. We compared the size of the prominent head-crest to foraging success at community-used feeding stations; we tested this relationship when competition was seasonally high, and when competition was seasonally low. We then experimentally decreased the number of feeders to increase competition (during the season of low-competition), and again tested whether male crest size predicted access to feeders. When competition was seasonally high, males with longer crests had greater access to feeders, but this pattern was not apparent when competition was seasonally low. When competition was experimentally increased, males with longer crests were again more successful at maintaining access to feeders. These findings provide evidence of a context-dependent status signaling system, where the status signal only mediates access to resources during periods of high competition. We discuss possible hypotheses for why the signaling system may not be functional, or detectable, during periods of low competition, including that competitors may interact less frequently and so have reduced opportunity for signaling, or that status signals are disregarded by receivers during periods of low competition because signalers are unlikely to escalate a contest into a fight. In any case, these results indicate that resource availability affects a status signaling system, and that the potential for status signaling persists in this system between seasons, even though such signaling may not be overtly present or detectable during periods of low competition.

## Introduction

Competition for limited resources is ubiquitous among social organisms, and highly competitive individuals often enjoy fitness advantages because they are able to garner priority access to resources [1]. However, establishing priority access can be costly, especially when contests are settled with physical fights [2]. Many animals have evolved conspicuous signaling traits that minimize the need for costly fights by relaying information on the signaler's resource holding potential (RHP). Such signals of status [3] have been described among diverse taxa including birds, lizards, fish, insects, and primates [4], and communicate various aspects of RHP, including phenotypic condition [5] and fighting ability [6]. Status signals influence competitive outcomes by modifying a receiver's decision to respond aggressively [7], and these signals have been shown to mediate access to resources across breeding and non-breeding contexts, including breeding territories [8,9], dominance position in stable groups [10] and winter flocks [11], and access to food [12–14]. Although status signals function to resolve agonistic interactions in various competitive contexts, it remains unclear whether the utility of these signals is influenced by the level of population-wide competition for access to resources, or if the utility of status signaling systems remains constant over time.

The degree to which individuals invest in resource defense is determined by optimizing the benefits associated with the resource against the costs of maintaining access against competitors [15]. Seasonal shifts in the value of a contested resource affect this balance [16]. Individuals can benefit by forgoing resource defense when the payoff of increased access does not outweigh the cost of defense. For instance, cessation of territorial defense can occur when there is a decrease in food availability because little is gained from defending rare resources [17], while in contrast, the presentation of supplemental food can lead to an increase in social competition and defense [18]. In addition, competition can be low in contexts when food is so abundant that it is non-limiting, and so diverting time and energy into defense does not increase access to resources [19]. These forms of strategic allocation to resource defense have been well studied [17,19–21], and results indicate a general pattern where individuals are more likely to invest in defense when a resource has greater value (often when it is neither too rare nor too abundant) [22,23]. The decision rule to match aggression to resource value was nicely demonstrated by Dearborn and Dearborn [24], who found that territorial hummingbirds defended feeding patches more aggressively when the caloric value of the sugar within flowers was experimentally increased.

In addition to resource value, the cost of aggression can also dramatically impact an individual's decision to engage in a fight. Models predict that the risk of injury and the expenditure of energy during a fight must be outweighed by the value of the resource [2,25]. Due to differences in RHP, some individuals face greater costs than others from fighting [1]. For example, in jumping spiders, smaller individuals with lower RHP are more likely to give up and invest less into contest duration than larger competitors [26], while in hermit crabs, contestants that lose a fight expend a greater proportion of energy reserves compared to winners [27]. Individuals with low RHP may also face greater physiological costs of aggression, such as high testosterone levels [28] and oxidative stress [29]. As a result, animals are expected to evaluate cost-benefit ratios based on their own RHP, as well as on perceived RHP of their competitor [25], and thus strategically invest into aggression accordingly [1,30]. When resources are highly valued, both those with high and low RHP are expected to actively compete for access to resources. However, individuals with high RHP are predicted to invest more into aggression because they can bear the cost of aggression, and as a result, dominant individuals generally acquire more food [31]. In contrast, where resources are of low value, neither dominants nor subordinates are expected to invest heavily in competition due to the costs of aggression and low payoff associated with winning [22].

When individuals strategically allocate investment into aggression, we expect the utility of a status signaling system, which mediates such aggressive interactions, to vary over the same time period. When resources are of high value, we expect that a status signaling system will function as expected, and that a status signal will determine access to resources. However, when competition is reduced, and the motivation to fight will be lost by both dominant and subordinate signalers, we expect status signals to become disassociated from access to resources. Indeed, a context-dependent relationship between ornamentation and signaler or receiver behavior has been described in other systems [32–34], as well as a context-dependent link between ornamentation and physiology [35] and life-history strategy [36]. These results suggest that signals should communicate relevant information only under specific contexts, or that receivers should only respond to signals when it is beneficial to do so. However, despite previous evidence that signals may function to communicate specific messages under particular conditions, little is known about how communication systems are altered by the competitive environment (for example, see [37]).

To test the hypothesis that a status signaling system is functional only during periods of high competition, we investigated seasonal changes in the role of the head crest, a putative status signal [38], in mediating access to food in the male black-crested titmouse (*Baeolophus atricristatus*), a species that experiences substantial changes in population density and competition for food over the annual cycle. We carried out two observational studies during different times of the annual cycle, and then experimentally reduced the amount available food by reducing the number of supplemental feeders. We compared the size of the prominent head crest (which is independent of body size, see Methods) of adult males to foraging success at community-used feeding stations. The first study was performed during a period of high population density (hereafter 'high-density') when territories are occupied by a breeding pair and multiple fledged offspring (which remain in residence for 1–6 months, [39]). This high-density period is a time when natural food resources on each territory are depleted by the large number of family-members in residence, and represents a time of the year when our supplemental seed-feeders are of great value (see Methods for further information on density and competition). Our second study similarly compared crest length to competitive outcomes, but this study occurred approximately 6 months later during the low population density period (hereafter 'low-density') of the annual cycle, when only a single pair was resident on each territory. Natural food resources are more abundant during this low-density period because each territory sustains the foraging habits of only two individuals (and also because of seed masts produced in the winter, there is more natural food during this period), and our supplemental seed-feeders are of less value. For our third approach, which was conducted immediately after the second study (also during the low-density period), we experimentally increased competition by reducing the number of feeders by approximately half—and again tested whether crest length predicted foraging success. These three approaches allowed us to test whether alterations in population-wide patterns of competition affect the utility of a status signal in mediating access to resources.

## Materials and methods

### General approach

**Fig 1:**
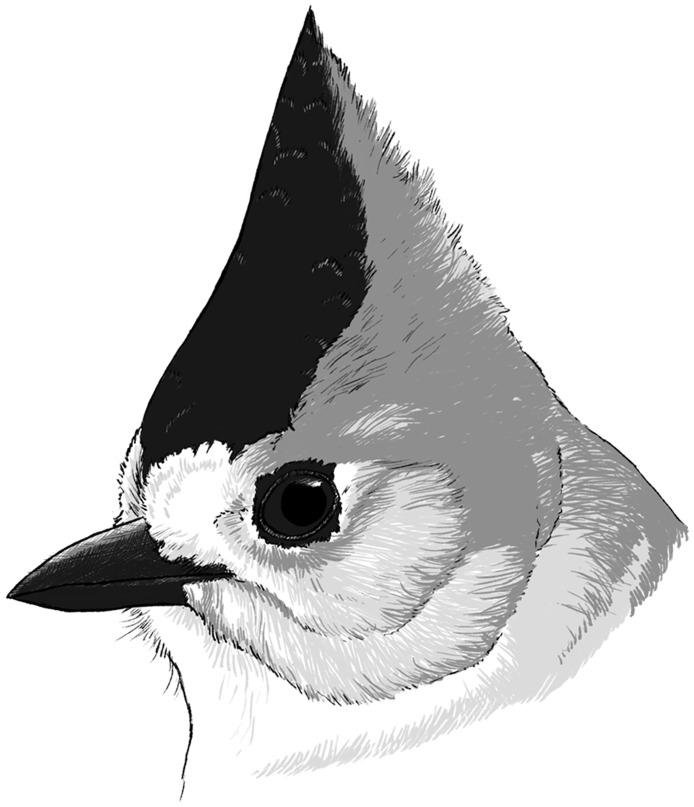
Crest of the black-crested titmouse, *Baeolophus atricristatus*.

The black-crested titmouse (*Baeolophus bicolor*) is a socially monogamous passerine with a conspicuous black crest (Fig 1). Although both sexes have an elaborate crest, we focused our study on the function of the male crest as a possible signal of status. Based on various lines of evidence, the titmouse crest is a good candidate to function as a status signal and communicate aggressive motivation or ability. First, the crest is prominently raised during agonistic interactions, linking dominance behavior to this ornamental trait [38]; second, the crest's black coloration is in stark visual contrast with other plumage, suggesting that selection has modified this trait to act as a signal; and third, research on the sister species, the tufted-titmouse, *Baeolophus bicolor*, has provided evidence that head plumage may be used as a signal of status during agonistic interactions [40].

Research was conducted on a ca 55 ha private ecological preserve in the hill-country of Texas, near Kendalia, TX (29°58’10.0”N, 98°31’30”W). The black-crested titmouse breeds between March and June and is a year-round resident in this region. Although core-territories are defended throughout the year, the species forages within larger home-ranges that overlap with multiple neighboring territories [39], and aggressive behavior is pronounced at artificial feeders located within overlapping home ranges (TGM personal observation).

Our study is divided into two periods that vary in population density. We conducted our first observational study between June 27-July 15 2013, which corresponds to the high-density period, when large nuclear family groups of 3–8 birds maintain territories (breeding pairs plus young produced that year). These groups can remain together for up to six-months [39]. Both the second and third study were conducted at the same study site approximately 7 months later between February 1–20 2014, which corresponds to the low-density period. This period refers to the time when only two birds reside on each territory, after young from that year have suffered juvenile mortality or have dispersed away from their natal territory. During the high-density period, we assume supplemental food is of substantially greater value than the low-density period because the large number of titmice on each territory are expected to deplete available natural food resources. Our preliminary findings support this assumption: each individual visited our supplemental feeders 1.5 times more often, on average, during the high-density period compared to the low-density period. The high value of supplemental feeders is likely compounded by a summertime (high-density period) decline in local arthropods [41], a main component of titmouse diet. Furthermore, acorns, which are a preferred food of titmice [38], mature in early winter in central Texas and their availability is substantially limited during summertime high-density period. Taken together, these observations support the assumption that there is greater competition between adult males on neighboring territories for access to supplemental food during the high-density period.

To measure access to supplemental feeders, we created an array of 11 feeders outfitted with RFID antennae and processors [42,43] spread within the core area of the 55ha study site. Feeders held black-oil sunflower seed and were modified to have only one feeding port which could be monopolized while an individual was foraging. Because this species removes a single seed from a feeder and flies to a nearby perch to process the seed, each recorded visit to the feeder represents the consumption of a single seed. Each time a tagged adult male titmouse fed from a feeder, its unique ID was stored as a data file, along with the time and date. Using RFID data, we quantified foraging success of each individual as the total number of foraging events to any feeder over the recording period.

### Capture and measurements of crest

Between May-June 2013, we captured birds using funnel traps placed around seed feeders. Upon capture, we measured size (tarsus) and body mass. Birds were banded with USGS metal band, individualized color bands, and a 12 mm PIT tag was attached to the color bands on one leg. Within our study site, we captured and tagged 15 adult males for the purpose of these studies.

Upon capture, crest length was measured digitally using standardized photos in which the crest was flattened and held parallel to the camera's sensor. A size scale was included in each picture and Image J [44] was used to measure the linear distance between the exposed culmen to the tip of the flattened crest. Repeatability of crest measurement was high (r = 0.98, N = 14 repeated measures, see below for explanation of sample size; [45]. Crest measurements from the original capture were used for all studies because there is not a systematic change in crest size between years (based in our larger longitudinal data set, unpublished data). All analyses use a raw measure of crest length because there was not a significant between crest length and body size (least-square regression: F_1,12_ = 0.14, p = 0.72).

### Study 1: Does crest length relate to resource acquisition when the value of supplemental feeders is high?

For study 1, which occurred during the high-density period in the summer, we compared crest length to our measures of access to resources. We monitored RFID data to assess foraging over 19 consecutive days (June 27-July 15, 2013). We began collecting RFID data after birds already had approximately 2 months experience with the feeders (i.e., feeders were available from mid-April), and so all birds in the area already had an opportunity to utilize the feeders. The RFID readers acquired data on foraging visits during all daylight hours (ca. 14 hrs per day). There were two occasions in which data from a single feeder was lost due to hardware damage by mammals, and so data were removed during this time-period from all 11 feeders so that all feeders recorded identical periods.

### Study 2: Does crest length relate to resource acquisition when the value of supplemental feeders is low?

For study 2, we again tested for a relationship between male crest size and access to resources by re-deploying RFID-equipped feeders during the low-density period before the breeding season (February 1–10, 2014). Before we began collecting RFID data, we replaced the 11 feeders for approximately one-month to allow all birds in the area to rediscover the resource. We conducted this second study as described above, and collected 10 days of baseline foraging data using the same 11 feeders outfitted with RFID readers.

### Study 3: When competition is experimentally elevated, are males with longer crests better able to maintain access to supplemental feeders?

After we established baseline usage of feeders during the low-density period in study 2, we conducted study 3: we experimentally increased competition by reducing the number of available feeders by approximately half (from 11 to 6 feeders). Immediately following study 2, we conducted Study 3 for 10 days, between February 11–20 2014. The removed feeders were evenly distributed spatially throughout the study site, and the removed feeders were balanced so that a similar number of feeders were removed from areas where dominant and subordinate males typically foraged. In other words, the number of short-crested individuals that lost their primary feeder was similar to the number of long-crested individuals that lost their primary feeder (as assessed by the feeder each individual most frequently visited during study 2). We removed the feeders at night so as not to interfere with foraging behavior. After feeders were removed, we collected RFID data in the same manner as listed above (i.e., during all daylight hours). There was one instance where a feeder was emptied of seed for 7 hours during the baseline period (study 2), and so data from all other feeders during these hours were removed from this dataset, and a corresponding period of data of equal duration, from the same time of day, and from the same day within the 10-day cycle, was removed from all feeders from the study 3 dataset.

To test whether crest length predicted maintenance to access to food when competition was increased, we calculated the percent reduction in foraging success for each individual upon the reduction of feeders (measured as difference between the number of foraging events during the experimentally reduced-feeder period minus the foraging events during baseline low-density period (from study 2), divided by total visits during the baseline low-density period. This measure represents an individual’s success at maintaining its own baseline level of access to feeders after competition was increased.

### Ethics statement

This study was carried out in strict accordance local and federal laws; vertebrate work was approved by an Institutional Animal Care and Use Committee (IACUC) (Permit Number: 090512_TM2). Handling of titmice was kept to a minimum, following recommendations of the Ornithological Council's Guidelines to the use of wild birds in research. All sampling procedures and experimental manipulations were approved as part of obtaining IACUC certification. The research on private property was conducted with permission of the owner.

### Statistics

General linear models were used to analyze foraging success data. Separate analyses we run for studies 1–3 to compare crest length to foraging success. Body size (tarsus) and body mass were included in all models to control for their possible effect on dominance. Factors were removed using backwards stepwise simplification if P > 0.10. All statistics were performed in JMP 13. Three tagged males were not observed after initial capture, and one male was removed from analyses because it was missing half of the distal feathers on the crest at the time of capture. Sample size was thus reduced from 15 to 11 males in study 1. For studies 2 and 3, sample size was further reduced from 11 to 7 males because 4 individuals disappeared after the first study. The males that disappeared had crest-lengths that were evenly distributed from the distribution of crest length (the following are the ranks of crest length of birds that were in study 1 that were later absent: 2nd, 4th, 9th, 11th), so their absence did not add a directional bias to our results. All research was performed following strict animal care guidelines and protocols were approved by IACUC (Trinity University 090512_TM2).

## Results

### Study 1: Does crest length relate to resource acquisition when the value of supplemental feeders is high?

**Fig 2:**
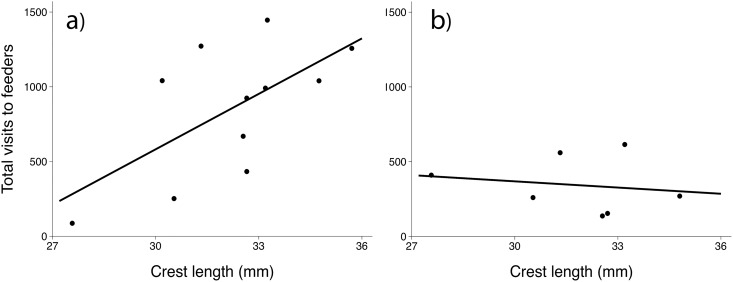
Relationship between male crest length and access to supplemental feeders, as assessed by total visits to feeders. (a) during the high-density period when the value of feeders is assumed to be high, and (b) during the low-density period when the value of feeders is assumed to be low.

We recorded a total of 9,412 foraging events from 11 male titmice over the 19-day high-density period. Each male foraged 855.6 ± 132.9 (mean ± SE) times throughout the study period, with a mean of 45.0 (range: 5–76) visits per bird per day. During this high-density period, crest length was positively correlated with total number of visits across all feeders (F_1,9_ = 5. 82, p = 0.039, R^2^ = 0.39, Fig 2A).

### Study 2: Does crest length relate to resource acquisition when the value of supplemental feeders is low?

During the 10-day baseline low-density period, we recorded 2,046 foraging events from 7 adult male titmice. Each male visited the feeders 343.7 ± 71.7 times during the study period, with a mean of 29.2 (range: 7–32) visits per bird per day. During this low-density period, there was not a significant relationship between crest length and total visits (F_1,5_ = 0.15, p = 0.72, Fig 2B).

### Study 3: When competition is experimentally elevated, are males with longer crests better able to maintain access to supplemental feeders?

During the 10-day winter reduced-feeder period, we recorded 1087 foraging events from 7 male titmice. Each male visited the feeders an average of 155.3 ± 41.0 times, with a mean of 15.5 (range: 3–17) visits per bird per day. Use of feeder resources decreased with the reduction in the number of feeders, with a significant decrease in total feeding events between baseline and the experimental removal of feeding stations (Wilcoxon signed rank: S = -14.0, p = 0.007). Individuals suffered a loss, on average, of 52.4% ± 9.8% of their baseline foraging success.

**Fig 3:**
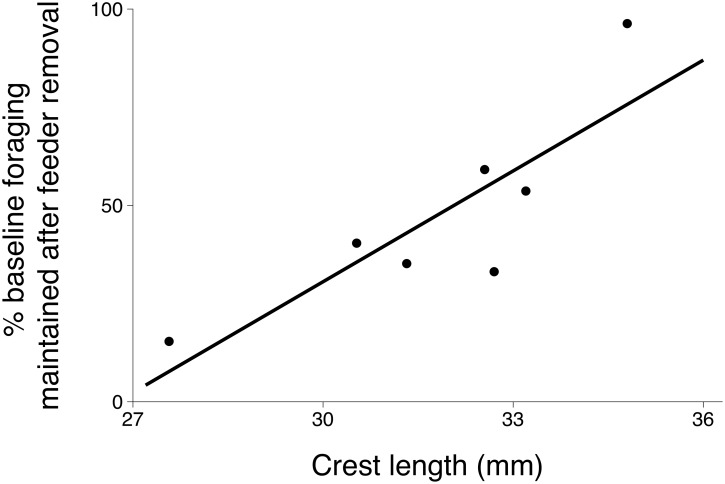
After experimental reduction in the number of supplemental feeders, the relationship between crest length and an individual's success at maintaining its own baseline level of access to feeders.

After the experimental reduction in the availability of food, crest length was significantly positively related to an individual's ability to maintain competitive status: individuals with larger crests maintained a larger percentage of their baseline feeding rate (F_1,5_ = 12.25, p = 0.017, R^2^ = 0.71, n = 7, Fig 3), indicating that longer crested males were less effected by the reduction in the number of feeders.

## Discussion

While status signals can effectively mediate conflict by reducing the need for costly fights [46], local competition has the potential to affect the utility of a status signaling system. We tested for a change in the relationship between a putative status signal and resource acquisition when there was a seasonal shift in the value of community-utilized feeders. When population density was high, we found that males with longer crests had greater access to feeders. This finding is consistent with the hypothesis that the crest functions as a status signal that mediates access to resources, and parallels other studies that show a relationship between status signals and access to food resources [13,47–49]. Furthermore, these results agree with previous research indicating that head-plumage functions as a signal of status in a sister species of titmouse [40]. In contrast, in our second study, which was performed when the population was less dense (and when feeders are likely to be of less value), we failed to detect a relationship between crest length and foraging success. Taken together, these results tentatively support the hypothesis that the utility of a status signaling system is dependent on the social environment, and that a signal of status may only mediate access to resources during periods of high competition.

When we experimentally increased competition during the low-density period by reducing the number of feeders, we found that longer crested males were better able to sustain their baseline foraging rates. That is, upon removal of approximately half of the feeders, longer crested males experienced only marginal reduction in access to feeders compared to those with shorter crests, which suffered up to 85% reduction from their baseline foraging rates. The results from this experimental test provides additional evidence that longer crested birds are more successful competitors, similar to results from study 1. By decreasing food availability, it appears that we increased competition and activated the signaling system so that long crested males were again better able to maintain access to resources. We note that the lack of a relationship between crest and foraging success during the low-density period is unlikely due to the reduced sample size (which was reduced after the disappearance of 4 males from the study site). The birds that disappeared before experimental study 3 were equally distributed between large- and short-crested individuals, so their absence did not directionally drive differences in the relationship between crest length and foraging success across studies. Furthermore, our failure to detect an effect of crest on foraging success in study 2 was unlikely due to low statistical power: studies 2 and 3 had the same sample size, yet there was sufficient statistical power in experimental study 3 to detect a moderate effect size. As such, we conclude that the relationship between crest length and access to food was reduced or absent during the period when the titmouse population density was low, when feeders were likely to represent a low-value resource.

The observation that male crest length is related to foraging success only during periods of high competition is consistent with our expectation that status signal systems will have an increased function when there is greater risk of fighting. Multiple avian species have been found to use status signals only during discrete times of the year, although in these examples, the trait is absent, or is noticeably diminished, when the signaling system is not fully functional. For example, the black throat badge of male house sparrows is partially obscured by white feather tips during the non-breeding season when signaling is reduced [50] and then becomes fully exposed during the breeding season when it is used as a signal [7]. Similarly, in the female American goldfinch, bill color changes from a dull brown to bright orange during the breeding season when the trait is used to signal status [13]. In contrast, titmice maintain their crest throughout the year, and so the seasonal breakdown of the signaling system requires additional explanation.

There are a number of hypotheses that could explain why a status signaling system would lose functionality during periods of low competition. The first possibility is that competitors may be simply less likely to interact during periods of reduced competition (e.g., if competitors are unlikely to visit a feeder simultaneously), and so have reduced opportunity to signal to one another. As a result, access to resources would not be mediated by a signal. A second possibility is that signalers may modulate the way their signal is displayed depending on the level of competition [51–54]. If a signal is only displayed when an individual is actively defending a resource, this would reduce the risk that the signaler would escalate a social interaction into an unnecessary fight. Among titmice, it is possible that the raising and lowering of the crest may convey information on current aggressive motivation and therefore dynamically reflect the readiness of a signaler to engage in resource defense. More research on the display of the crest is required to address this hypothesis. A third possible explanation for the lack of relationship between signal expression and access to resources focuses on the receiver of the signal: receivers may disregard information encoded in a status signal when a signaler is unlikely to escalate a contest into a fight. In titmice, an individual may fail to avoid a longer-crested competitor because it assess that the signaler is unlikely to actively defend a resource—which would be expected during periods of low competition when the cost of a potential fight (to the signaler) is likely to outweigh the benefits of resource acquisition [23]. Theoretical models predict that signals of status serve as honest reflections of fighting ability on average [46,55], but these models assume that signalers generally back up their signal with an in-kind aggressive response [56]. In cases where motivation to be aggressive varies over time, we propose that status signaling systems can cease to function during times of low competition. For such a signaling system to remain stable, however, receivers would need a way to assess the appropriate times to pay attention to signals, and when to disregard them. One way this could be done is if individuals evaluate population-wide levels of competition and adjust the way they attend to status signals accordingly. More research is needed to assess how receivers in a status signaling system vary their behavior with fluctuations in the competitive environment (see, [37].

One limitation of our study is that we did not conduct behavioral observations to confirm that long-crested males actively exclude short-crested competitors. As such, it is possible that the pattern we detected (that individuals with longer crests are better able to take advantage of a supplemental feeders) may not relate directly to signaling. For instance, males with longer crests may win fights with short crested birds over access to supplemental feeders without the use of a signal. However, theory predicts that fights will be relatively rare if they can be avoided through signaling [57], and we would find it highly unusual for a prominent trait, like the titmouse crest, to be linked to competitive success, yet not function as a signal that influences competitive decisions. An alternative explanation for our results is that the feeders represent an inconsequential resource that do not induce competition, and long crested birds feed from them more frequently for unexplored reasons. Although, this alternative must be considered, the heavy use of our feeders by territory owners that range from across the study site suggests that these feeders are valuable resource—possibly because the feeders provide caloric-rich seeds or because their use allows reduced energy spent foraging for other more dispersed resources.

Our results indicate that resource availability affects a status signaling system, and that the potential for status signaling persists in this system between seasons, even though such signaling may not be overtly present or detectable during periods of low competition. Because our results provide evidence that a status signal is actively used during only part of the year, we urge future research to consider the possibility that a signaling system may not be functional during the time-period under investigation (also see [58], as this perspective may help explain previous findings indicating inconsistencies in the relationship between signal value, aggression, and testosterone [52]. Furthermore, considering status signals in the wider context of social selection—an umbrella term that refers to competition for both mate-based (sexual selection) and non mate-based resources—could help tease apart the context-dependent nature of social signals [59–62]. Finally, we urge future research to test for fluctuations in the utility of a status signaling system across variable competitive environments.
